# Response surface methodology and repeated-batch fermentation strategies for enhancing lipid production from marine oleaginous *Candida parapsilosis* Y19 using orange peel waste

**DOI:** 10.1186/s12934-024-02635-3

**Published:** 2025-01-10

**Authors:** AbdAllah M. Matouk, Gadallah M. Abu-Elreesh, Mohamed Ali Abdel-Rahman, Said E. Desouky, Amr H. Hashem

**Affiliations:** 1 Botany and Microbiology Department , Faculty of Science, Al-Azhar University, Nasr City, Cairo 11884 Egypt; 2 Engineering and Biotechnology Research Institute (GEBRI), City of Scientific Research and Technological Applications (SRTA-City), New Borg Al-Arab City, Alexandria 21934 Egypt

**Keywords:** Oleaginous yeast, Orange peels lipid production, Taguchi design, Repeated batch fermentation, *Candida parapsilosis*

## Abstract

Oleaginous yeasts are considered promising sources for lipid production due to their ability to accumulate high levels of lipids under appropriate growth conditions. The current study aimed to isolate and identify oleaginous yeasts having superior ability to accumulate high quantities of lipids; and enhancing lipid production using response surface methodology and repeated-batch fermentation. Results revealed that, twenty marine oleaginous yeasts were isolated, and the most potent lipid producer isolate was *Candida parapsilosis* Y19 according to qualitative screening test using Nile-red dye. Orange peels was used as substrate where *C. parapsilosis* Y19 produced 1.14 g/l lipids at 23.0% in batch fermentation. To enhance the lipid production, statistical optimization using Taguchi design through Response surface methodology was carried out. Total lipids were increased to 2.46 g/l and lipid content increased to 30.7% under optimal conditions of: orange peel 75 g/l, peptone 7 g/l, yeast extract 5 g/l, inoculum size 2% (*v/v*), pH 5 and incubation period 6 d. Furthermore, repeated-batch fermentation of *C. parapsilosis* Y19 enhanced lipid production where total lipids increased at 4.19 folds (4.78 g/l) compared to batch culture (before optimization). Also, the lipid content was increased at 1.7 folds (39.1%) compared to batch culture (before optimization). Fatty acid profile of the produced lipid using repeated-batch fermentation includes unsaturated fatty acids (USFAs) at 74.8% and saturated fatty acids (SFAs) at 25.1%. Additionally, in repeated-batch fermentation, the major fatty acid was oleic acid at 45.0%; followed by linoleic acid at 26.0%. In conclusion, *C. parapsilosis* Y19 is considered a promising strain for lipid production. Also, both statistical optimizations using RSM and repeated-batch fermentation are efficient methods for lipid production from *C. parapsilosis* Y19.

## Introduction

The primary sources of oils and fats in the world are derived from fish, animals, plants (especially vegetable oil), and microorganisms [1]. Microorganisms that classified as oleaginous microorganisms are those that possess the capacity to generate and store 20–80% of their entire biomass as intracellular lipids [2]. Using microbial lipids, commonly known as single-cell oils, is one method for improving oil output. These oils can serve as a feasible substitute feedstock for biodiesel production and as an alternative path to a bio-based economy [3]. Oleaginous microorganisms, encompassing microalgae, bacteria, fungi, and yeast, have the capacity to generate lipids in their cellular compartment exceeding 20% (*w/w*) of the total lipid content, as determined by cell dry weight [4]. Compared to plant and fish oil production, microbial oil production offers a number of benefits, such as quick development, high oil content, and high oil composition quality [5, 6]. Most lipids produced by oleaginous microorganisms have an unbranched carbon chain length of four to twenty-eight. The type of hydrocarbonated chain determines whether the fatty acids are saturated or unsaturated, and the quantity of double bonds determines whether they are monounsaturated or polyunsaturated (MUFA and PUFA) [4].

Oleaginous yeasts represent a distinct assemblage of microorganisms characterised by their exceptional capacity to a mass substantial amounts of intracellular lipids or oils. This property makes them highly attractive for industrial-scale production of lipids, which have a diverse range of applications in the biofuels, oleochemicals, and animal feed industries [7]. Under optimal growth conditions, oleaginous yeasts have the potential to accumulate up to 70% of their total dried cell weight in lipids. This is significantly higher than the typical lipid content of 5–20% found in regular, non-oleaginous yeast species. This exceptional lipid-producing ability is attributed to the specialized metabolic pathways present in oleaginous yeasts [8]. The key to the success of oleaginous yeasts in lipid production lies in their ability to efficiently convert various carbon sources, such as glucose, xylose, glycerol, and even waste streams, into lipids. This is achieved through a series of metabolic processes that channel the carbon flux towards the synthesis and accumulation of triacylglycerols, the primary storage lipids in these organisms [9, 10]. Oleaginous yeasts are highly advantageous over filamentous fungus and algae because of their capacity to grow quickly on a wide range of substrates, their simplicity of cultivation, and their great responsiveness to process scaling-up [11]. The kind and quantity of lipid generated are significantly impacted by the fungus species, growth conditions, and nutritional requirements [1]. Therefore, improving these conditions is a crucial first step since it leads to increased lipid production at a cost that is affordable for continued industrialization [12]. The fatty acid profile of single cell oils varies depending on the type of microbe, which makes them ideal for a wide range of industrial applications [13]. For example, human consumption and a few worthwhile industrial uses, such as the production of biodiesel, paints and coatings, detergents, cleaning supplies, and cosmetics [14].

Agricultural wastes can be used as low-cost substrates for oleaginous fungi for lipid production [15–17]. In accordance with the Food and Agriculture Organization of the United Nations (FAO, 2023), Brazil continues to hold the position of being the foremost global producer of oranges, accounting for around 35% of the world’s overall production. This production was predicted to be over 50 million metric tons in the year 2022. The orange, which is the major citrus fruit, is among the top five key fruit commodities that dominate the global fruit industry. Approximately 40–60% of oranges designated for juice production are ultimately disposed of as garbage, encompassing the peel, segment membrane, and seed [18]. Citrus peel is the main component among these wastes, making up about 44% of the weight of the fruit mass [19]. Citrus waste is used in a variety of processes, including the synthesis of fiber, pectin, flavonoids, and animal feed [20]. However, a sizable portion of this waste is still disposed of annually [21]. This results in issues with the environment and economy, including increased transportation costs, a shortage of disposal sites, and an accumulation of material with a high organic content [22].

Consequently, it is critical to transform these wastes into products with added value by either utilizing extraction and purification procedures to recover bioactive components or utilizing microbial fermentations to use these wastes as a substrate for the synthesis of green chemicals [23]. Thus, it would be ideal to find more sustainable and efficient ways to use orange peel waste.Numerous papers discuss the use of orange peel as a substrate for the manufacture of SCO.

The utilization of orange peel as a substrate for the production of lipids by oleaginous yeasts is a promising approach that can contribute to the development of more sustainable and circular bioeconomy models [24]. Orange peel is an abundant agricultural waste product generated in large quantities by the citrus processing industry, making it an attractive and low-cost feedstock for microbial lipid production [25]. The composition of orange peel consists predominantly of cellulose, hemicellulose, pectin, and simple sugars, including glucose and fructose. These carbohydrate compounds can function as viable carbon sources for the proliferation and lipid buildup of oleaginous yeasts. However, the intricate structure and composition of orange peel necessitate pretreatment and hydrolysis procedures in order to facilitate the accessibility of sugars for microbial utilization [26]. There is not enough knowledge on marine yeasts and their lipid contents as dietary supplements when it comes to lipid production. The overall lipid content and the composition of cellular fatty acids in a particular yeast strain can be greatly impacted by adjusting growing circumstances, such as temperature, pH, and other minerals in addition to carbon input [27]. Thus, the objective of this study was to isolate and identify oleaginous yeasts capable of accumulating substantial amounts of lipids. Additionally, it aimed to utilize orange peel as a growth substrate for these yeasts and to optimize lipid production through Taguchi design. This substrate has not been widely explored in the previous literature, especially for the oleaginous yeasts. Moreover, to enhance the lipid production using repeated-batch fermentation.

## Materials and Methods

### Sampling and isolation of yeasts

Samples of seawater were collected from Abu Qir Bay, located in the Mediterranean Sea near Alexandria, Egypt. The collected specimens were placed in a refrigerator at 4 °C and transferred to the laboratory using sterile procedures. Isolation of yeast on Yeast extract, Peptone, Dextrose (YPD) medium (1% yeast extract, 2% peptone, 2% dextrose) was carried out according to method used by Yu et al. [28] with minor modifications. YPD medium supplemented with 0.01% (*w*/*v*) ampicillin (Sigma-Aldrich, St. Louis, MO, USA) and 0.01% (*w*/*v*) streptomycin (Sigma-Aldrich) to prevent bacterial growth was prepared and sterilized. Seawater samples were transferred to surface of YPD plates, then incubated at 25 ^o^C for 7 days. During this period, individual yeast colonies were picked and transferred to fresh YPD to isolate pure cultures. Yeast strains were suspended in 30% glycerol (*v*/*v*) and stored at − 80 °C.

### Screening for lipid production by yeast isolates

All yeast isolates were screened qualitatively for lipid accumulation using a Nile-red staining assay [29, 30]. The yeast biomass was incubated with 0.5 ml of 0.1 mM phosphate buffer saline (PBS) pH 7.4 and 0.05 ml of Nile-red solution in the absence of light. After a duration of 30 min, a thin layer was formed on a pristine glass slide and left to dry in the air. Fluorescence microscopy (Olympus BX 40) was utilized for the examinations.

### Identification of the most promising lipid-producing isolate

#### Morphological identification

The yeast’s morphological properties were examined and recorded. The morphological characteristics include both macroscopic characteristics of the colonies, such as colony color and appearance, as well as microscopic examination [31, 32]. The light microscope was used to observe the microscopic features. YPD colonies were selected and streaked onto CHROM agar^®^ plate to detect the species of *Candida*. The plates were then incubated at 37 °C for 48 h.

#### Molecular identification

The preparation of yeast cells involves the resuspension of 50–100 mg in isotonic buffer. The following step involves the addition of 750 µl of BashingBead™ Buffer to the mixture in a ZR BashingBead™ Lysis Tube. For a minimum of five minutes, the tube is bead beater-secured and processed at maximal speed. The vial is, subsequently, centrifuged at 10,000 *xg* for 1 min. The supernatant is centrifuged at 8,000 *xg* for 1 min after being transferred to a Zymo-Spin™ III-F Filter. The filtrate is supplemented with Genomic Lysis Buffer, and 800 µl of the mixture is transferred to a Zymo-Spin™ IICR Column. The process is repeated, and the column is filled with 200 µl of DNA Pre-Wash Buffer and 500 µl of g-DNA Wash Buffer. The column is then centrifuged at 10,000 *xg* for 1 min after each addition. The study used 28S rRNA primers for PCR, with a reaction volume of 50 *µ*L. The primers were annealed at 50 °C for one minute, denaturated at 95 °C for one minute, and elongated at 72 °C for two minutes. The final extension stage was carried out for 10 min at 72 °C. A negative control was sterile deionized water. A red safe dyed agarose gel was made using 1X TBE buffer. The bands produced were observed under UV light. PCR products were stored at 20 °C before use [33, 34]. The ABI 3730xl sequencer was used to sequence the PCR product.

#### Orange peels used

Orange peel waste used in this study was collected from Nasser Agricultural Secondary School in Damanhur, Behera Governorate, Egypt. The percentages of carbon, hydrogen and nitrogen was determined for the orange peel waste using elemental analyzer (Flash 2000 thermo scientific).

Boiling orange peels is a straightforward and cost-effective method to prepare them for utilization as a carbon source. Boiling orange peels offers a simple and economical method for preparing them as a carbon source. The orange peels undergo a washing and drying process at a temperature of 50 °C to remove contaminants and enhance the ability of solvents to interact with the peels. The peels are pulverized into a fine powder to increase the surface area, which enhances the extraction process during boiling. The powder is simmered in water for a duration of 15 to 30 min to extract the components that are soluble in water. Once the extract has cooled, it is separated from the peels using a cheesecloth filter [35].

#### Dry weight determination, lipid extraction, and lipid quantification

After the incubation period, the mycelia from the culture broth were collected in triplicates using a simple filtration process with Whatman No.1 filter paper. The dry biomass weight was measured using gravimetric analysis and reported in grams per liter (g/l) as described by Devi et al. [36]. Lipid extraction was performed following the method described by Bligh, Dyer [37]. In this method, 50 ml of cultured cells were subjected to centrifugation at 5000 *xg* for 5 min. The resulting pellets were then washed twice with 50 ml of distilled water. Subsequently, the pellets were added to 10 ml of 4 M HCl and incubated at 60 °C for two hours to break down the cell wall of the yeast strains. The acid-hydrolyzed solution mentioned above was continuously agitated at room temperature using 20 ml of solvents (a mixture of chloroform and methanol in a ratio of 2:1, volume to volume) for 3 h. Subsequently, the solution underwent centrifugation at a force of 2000 times the acceleration due to gravity for a duration of 5 min at room temperature to segregate the organic lower phases from the aqueous upper phase.

The lipids included in the organic lower phase were separated by filteration using filter paper and subsequently dried in an oven at a temperature of 60 °C until a consistent weight and dry biomass were obtained. Methanol was employed to destabilize the phospholipid layer, facilitating the dissolution of lipid droplets and subsequent recovery of the lipids by the non-polar solvent, chloroform. The weight of the dehydrated lipid was determined using the gravimetric technique. The total lipid yield, represented as a percentage of the total weight, was determined using the following equation:

$$\begin{aligned} &Total\,  lipid \,extraction\, \\ & \quad yield\, (\% )   &=  \frac{{{\text{weight of lipid extracted (g)}}}}{{{\text{weight of yeast biomass}}}}{\text{ }} \times {\text{100}} \\ \end{aligned}$$

#### Optimization of lipid production using a statistical design

The experimental data obtained were subjected to analysis using Minitab 18 Statistical Software. A statistical optimization approach was employed to choose six criteria for media analysis. A total of 25 experiments were done in accordance with the Minitab 18 design. All tests were performed in triplicate, and the resulting experimental data were reported as the averages of three replicates. The use of the Taguchi design was implemented in order to optimize the factors that impact lipid synthesis, encompassing the utilization of orange peel as a carbon source, nitrogen supply, inoculum size, incubation length, and pH adjustments. Table 1 presents a comprehensive overview of various factors influencing lipid production. These factors encompass differences in orange peel concentrations (50, 75, 100, 125, and 150 g/l), inoculum sizes (2, 4, 6, 8, and 10% *v/v*), initial pH values (5, 6, 7, 8, and 9), incubation period (2, 4, 6, 8, and 10 days), peptone concentrations (1, 3, 5, 7, and 9 g/l), and yeast extract concentrations (1, 2, 3, 4, and 5 g/l).

**Table 1:** Different factors and their levels for lipid production

Factor	Level 1	Level 2	Level 3	Level 4	Level 5
Orange Peel (g/l)	50	75	100	125	150
Incubation period (days)	2	4	6	8	10
Initial pH value	4	5	6	7	8
Yeast extract (g/l)	1	2	3	4	5
Peptone (g/l)	1	3	5	7	9
Inoculum size (%, *v/v*)	2	4	6	8	10

#### Repeated batch fermentation

Repeated batch fermentations of *C. parapsilosis* Y19 for lipid production was carried out according to method used by Alrefaey et al. [38]. A 500 mL flask with a working capacity of 100 mL was utilized, employing optimum medium components and conditions in accordance with the Taguchi design scheme. The fermentations were performed utilizing a concentration of 75 g/l for orange peel, 5 g/l for yeast extract, 7 g/l for peptone, a temperature of 30 °C, an inoculum size of 2% (*v/v*), and an initial pH of 5.0. Following each run, the medium underwent centrifugation at 5000 *rpm* for 10 min. The cells derived from each batch (run) were used for tinoculating the subsequent batch (run).

#### GC-MS analysis

The obtained lipids were treated with methanolysis, as described by Amaretti et al. [39], with the objective of transforming the fatty acids into fatty acid methyl esters (FAMEs). The resultant FAMEs were further assessed utilizing a Gas Chromatography 1310-ISQ mass spectrometer manufactured by Thermo Scientific, located in Austin, TX, USA. The spectral data was obtained within the mass-to-charge ratio (*m/z*) range of 40 to 1000, employing the full scan mode. An adjustment was made to the temperature of the ion source to 200 °C. The identification of the components was achieved by comparing their retention lengths and mass spectra with datasets from the WILEY 09 and NIST 11 mass spectral databases.

## Results and Discussion

### Isolation and screening of oleaginous yeasts

**Fig. 1:**
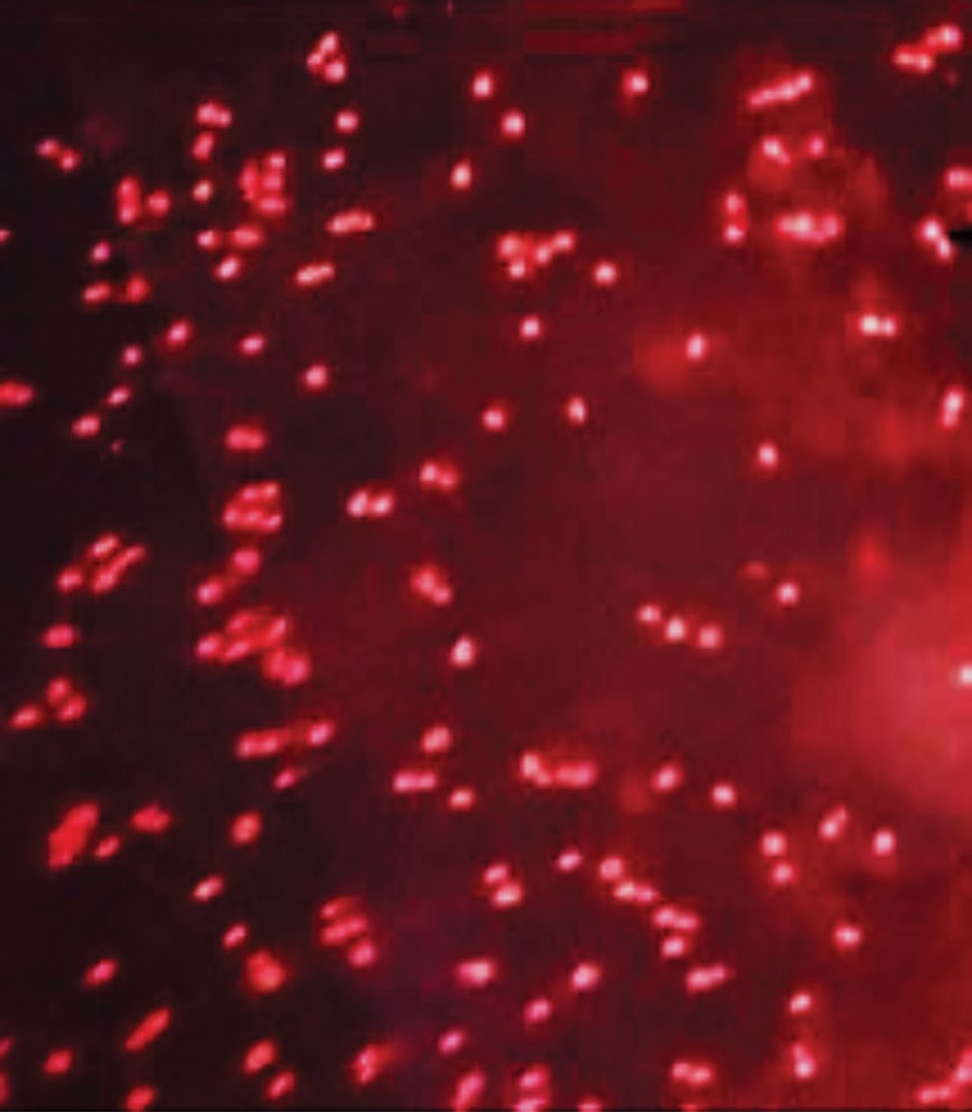
Lipid bodies produced by yeast isolate Y19 under fluorescence microscopy using Nile-red dye

Twenty yeast isolates Y1-Y20 were obtained from seawater marine sample, Abu Qir, Alexandria, Egypt. Nile Red is a widely used fluorescent dye for the detection and visualization of lipid bodies (lipid droplets) in cells, many reported articles used of Nile-red fluorescent dye for detection of lipid accumulation [29, 40, 41]. In the current study, qualitative screening of all yeast isolates for lipid accumulation was carried out according to detection of lipid bodies using fluorescence microscope. Results revealed that, isolate Y19 is the highest for lipid accumulation, where Fig. 1 shows presence of frequent lipid bodies that indicates high lipid accumulation by this isolate. Thus, isolate Y19 was selected for the further experiments. Kraisintu et al. [42] reported that, *Rhodosporidium toruloides* DMKU3-TK16 was the highest for lipid accumulation among other yeast according to Nile-red dye method. Vinarta et al. [43] used Nile-red fluorescence method for lipid accumulation of yeasts isolated from Antarctica where observed lipid bodies in most yeast isolates under fluorescence microscope.

### Identification of yeast isolate Y19

**Fig. 2:**
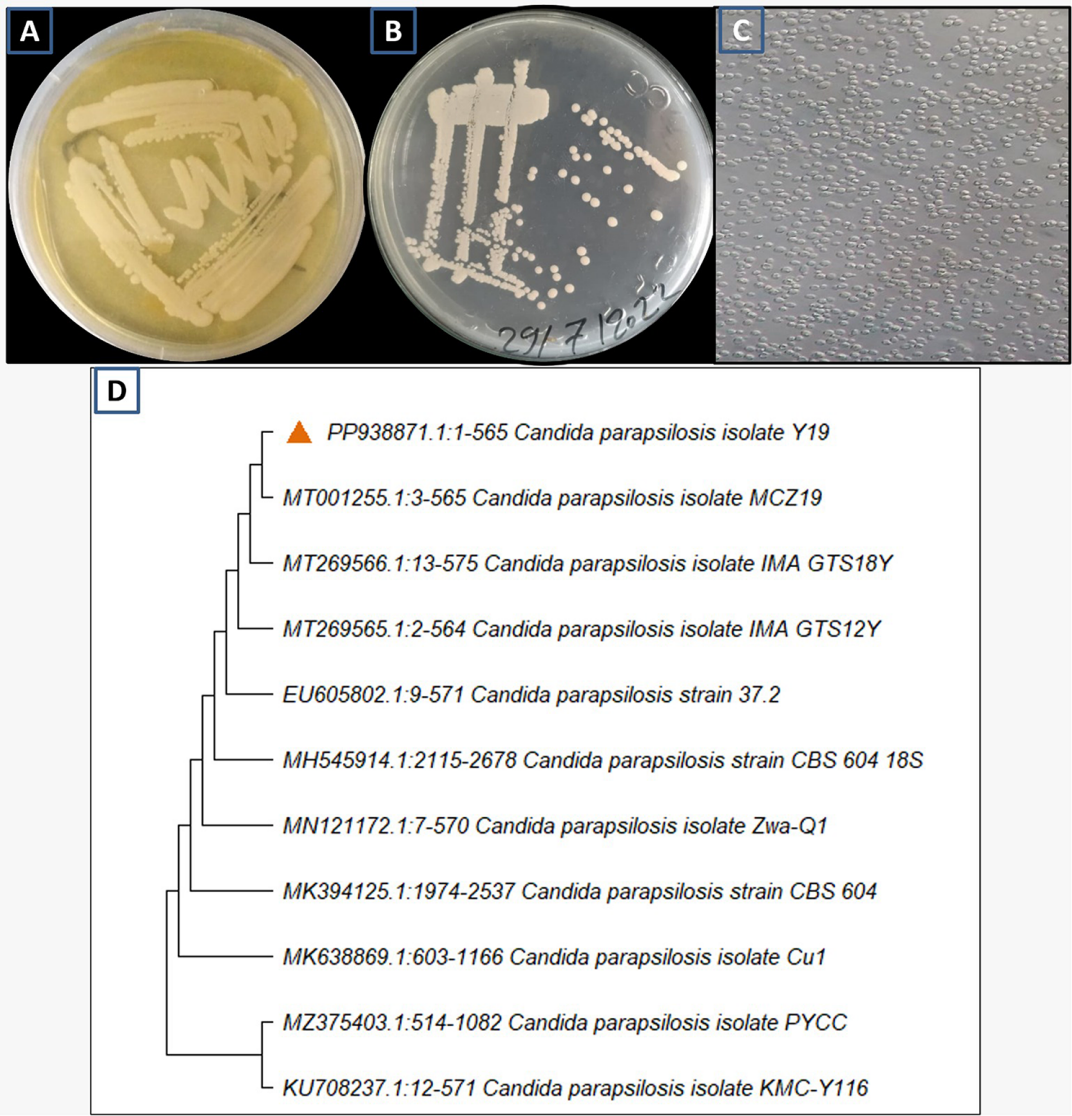
Routine identification (Surface growth of isolate Y19 on YPD (**A**), chrom agar (**B**) and isolate Y19 under light microscope 800X (**C**)) and phylogenetic tree of *Candida parapsilosis* isolate Y19 (**D**)

The yeast isolate Y19 exhibited both macroscopic and microscopic characteristics that confirmed its high potency. Y19 displayed a yellowish-white hue on YPD, a faint to glossy appearance, and an oval to spherical form with bipolar budding, as depicted in Fig. 2A&C. Also, the colonies appeared white to creamy in color on CHROM agar^®^, this confirms the isolate Y19 is resemble to *Candida parapsilosis* (Fig. 2B).

In order to validate the morphological identification, the present study conducted a molecular identification of the yeast isolate Y19 by analysing the 28S rRNA gene sequence. According to BLAST analysis, the selected yeast isolate Y19 was similar to *Candida parapsilosis* isolate MCZ19 (MT001255.1) with 99.4%. Then, *Candida parapsilosis* isolate Y19 was deposited in Gene-bank with accession number PP938871.1 (Fig. 2D). Many studies reported *Candida parapsilosis* has ability to accumulate lipids [44–46]. Nguyen and Nosanchuk [45] reported that, *C. parapsilosis* has some enzymes such as fatty acid desaturase (OLE1) and fatty acid synthase (FAS2) which enable the microorganism to accumulate lipids.

### Substrates for lipid production from *C. parapsilosis* Y19

Common oleaginous species produce high quantities of lipids include *Y. lipolytica*,* R. glutinis*, *L. starkeyi*, and *C. curvatus* [47–51]. Previous literatures reported that *Candida* species are oleaginous yeasts having the ability to produce lipids more than 20% of their cell dry weight [52–55]. In this study, carbon, hydrogen and nitrogen were determined for orange peel waste, where results illustrated that the percentages were 46.5, 11.0 and 2.5%, respectively. In the current study, *C. parapsilosis* Y19 was grown on glucose 60 g/l and pretreated orange peel 100 g/l as an alternative cheap carbon source for lipid production as shown in Table 2. Results revealed that, lipid content of *C. parapsilosis* isolate Y19 grown on glucose was 25.7% and total lipids was 1.84 g/l indicating that *C. parapsilosis* isolate Y19 is oleaginous yeast. On the other hand, *C. parapsilosis* isolate Y19 grown on pretreated orange peel produced lipids 1.14 g/l with lipid content of 23.0%. Oleaginous yeasts are a group of yeast species that have the ability to accumulate more than 20% of the yeast’s dry cell weight [56]. Thus, *C. parapsilosis* isolate Y19 is considered oleaginous where could accumulate lipids more than 20% of cell dry weight in the case of glucose and orange peel. Orange peel, an abundant agricultural waste product, has been explored as a potential low-cost substrate for the cultivation of oleaginous fungi. These fungi, such as *R. toruloides* NRRL 1091 and *C. laurentii* UCD 68–201 have the ability to convert the sugars and other nutrients present in orange peel into valuable lipids that can be used for biofuels, oleochemicals, and other applications [57, 58].

**Table 2:** Lipid production by *C. parapsilosis* Y19 strain on glucose and orange peel

Carbon source	Biomass (g/l)	Total Lipid (g/l)	Lipid content %
Glucose	7.16	1.84	25.7%
Orange peel	4.95	1.14	23.03%

The metabolism of lipid production in yeasts involves several key biochemical pathways that facilitate the conversion of carbon sources into fatty acids and triglycerides. Yeasts primarily utilize fatty acid synthesis pathways, where acetyl-CoA serves as a central metabolite. During growth on substrates such as orange peel, *C. parapsilosis* can efficiently convert sugars into acetyl-CoA through glycolysis and the citric acid cycle [59]. The accumulated acetyl-CoA is then directed towards fatty acid synthesis, which occurs in the cytoplasm via the fatty acid synthase complex. Additionally, the regulation of lipid production is influenced by environmental factors, including nutrient availability and the presence of specific carbon sources [59–61].

### Statistical optimization of lipids production by *Candida parapsilosis* using Taguchi design

Oleaginous yeasts can retain and accumulate cellular lipids if cultivation nutrients run out, provided that the carbon source remains available. As a result, the carbon is taken up by the yeasts and is assimilated into the cells as lipid droplets [56]. The Taguchi design approach is a powerful tool for optimizing lipid production in various systems [62]. By applying this statistical method, researchers can systematically identify the critical parameters that influence lipid yield and determine the optimal combination of these factors [63]. This approach typically involves conducting a series of experiments based on an orthogonal array, which allows for the efficient evaluation of multiple variables simultaneously [64]. The Taguchi method enables the identification of the most significant factors affecting lipid production, such as nutrient concentrations, pH, temperature, and cultivation time, and the determination of their optimal levels, leading to enhanced lipid yields [65, 66].

**Fig. 3:**
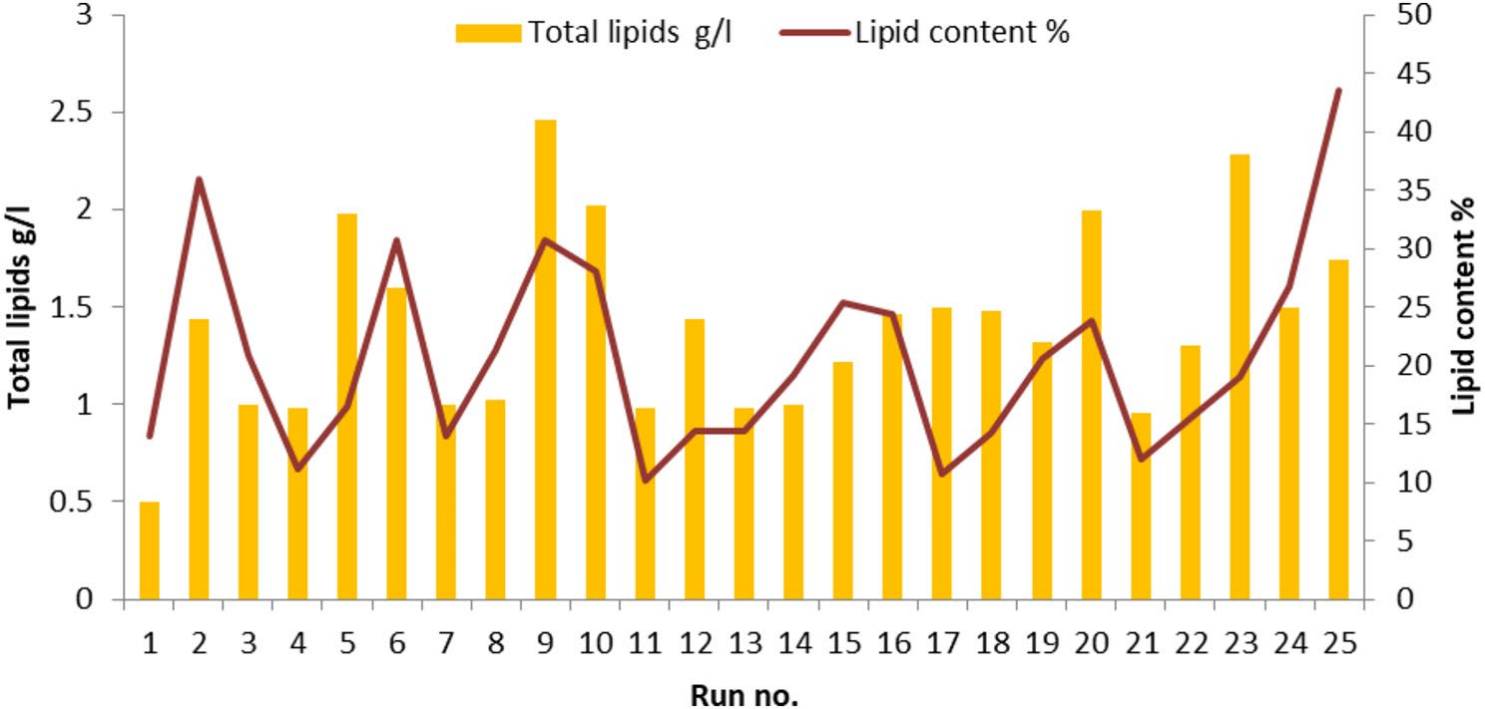
Effect of orange peel, peptone, yeast extract, inoculum size, pH and incubation time on lipid production by *Candida parapsilosis* Y19 using Taguchi design

A L25 orthogonal array was designed for optimizing various growth factors for lipid production by *C. parapsilosis* isolate Y19 as shown in Table 1. Six factors; orange peel, peptone, yeast extract, inoculum size, pH and time were included in the Taguchi design to detect the best level for each factor in one experiment. Previous studies reported that, the incubation temperature at 30 °C is the optimum for lipid production from yeasts [67, 68]. The growth temperature of oleaginous microorganisms affected the fatty composition and degree of saturation of the accumulated TAGs, the lipid concentration and fatty acid profile [69]. Fermentation at high temperatures produces more saturated than unsaturated fatty acids, while incubation at low temperatures generally favored the production of unsaturated fatty acids [70, 71]. Glucose as a simple and expensive carbon source was replaced by low value orange peel waste to reduce the production costs. Table 3; Fig. 3 illustrates that run no. 9 was the best for lipid production, and this run supplemented with an orange peel concentration of 75 g/l at initial pH value of 5.0, an incubation period of 6 days, inoculum size 2% (*v/v*), yeast extract 5 g/l, and peptone 7 g/l with fixed factor incubation temperature 30˚C which produced lipid 2.46 g/l, dry biomass 8 g/l, and lipid content 30.7%.

**Table 3:** Taguchi design of the selected factors for lipid production by *C. parapsilosis* Y19 strain

Run no.	Orange Peel (g/l)	Peptone (g/l)	Yeast extract (g/l)	Inoculum size (%, *v/v*)	Initial pH	incubation period (days)	Biomass Conc. (g/l)	Total lipids (g/l)	Lipid content %
1	50	1	1	2	4	2	3.60	0.50	13.89
2	50	3	2	4	5	4	4.00	1.44	36.00
3	50	5	3	6	6	6	4.80	1.00	20.83
4	50	7	4	8	7	8	8.80	0.98	11.14
5	50	9	5	10	8	10	12.00	1.98	16.50
6	75	1	2	6	7	10	5.20	1.60	30.77
7	75	3	3	8	8	2	7.20	1.00	13.89
8	75	5	4	10	4	4	4.80	1.02	21.25
9	**75**	**7**	**5**	**2**	**5**	**6**	8.00	**2.46**	30.75
10	75	9	1	4	6	8	7.20	2.02	28.06
11	100	1	3	10	5	8	9.60	0.98	10.21
12	100	3	4	2	6	10	10.00	1.44	14.40
13	100	5	5	4	7	2	6.80	0.98	14.41
14	100	7	1	6	8	4	5.20	1.00	19.23
15	100	9	2	8	4	6	4.80	1.22	25.42
16	125	1	4	4	8	6	6.00	1.46	24.33
17	125	3	5	6	4	8	14.00	1.50	10.71
18	125	5	1	8	5	10	10.40	1.48	14.23
19	125	7	2	10	6	2	6.40	1.32	20.63
20	125	9	3	2	7	4	8.40	2.00	23.81
21	150	1	5	8	6	4	8.00	0.96	12.00
22	150	3	1	10	7	6	8.40	1.30	15.48
23	150	5	2	2	8	8	12.00	2.28	19.00
24	150	7	3	4	4	10	5.60	1.50	26.79
25	150	9	4	6	5	2	4.00	1.74	43.50

**Fig. 4:**
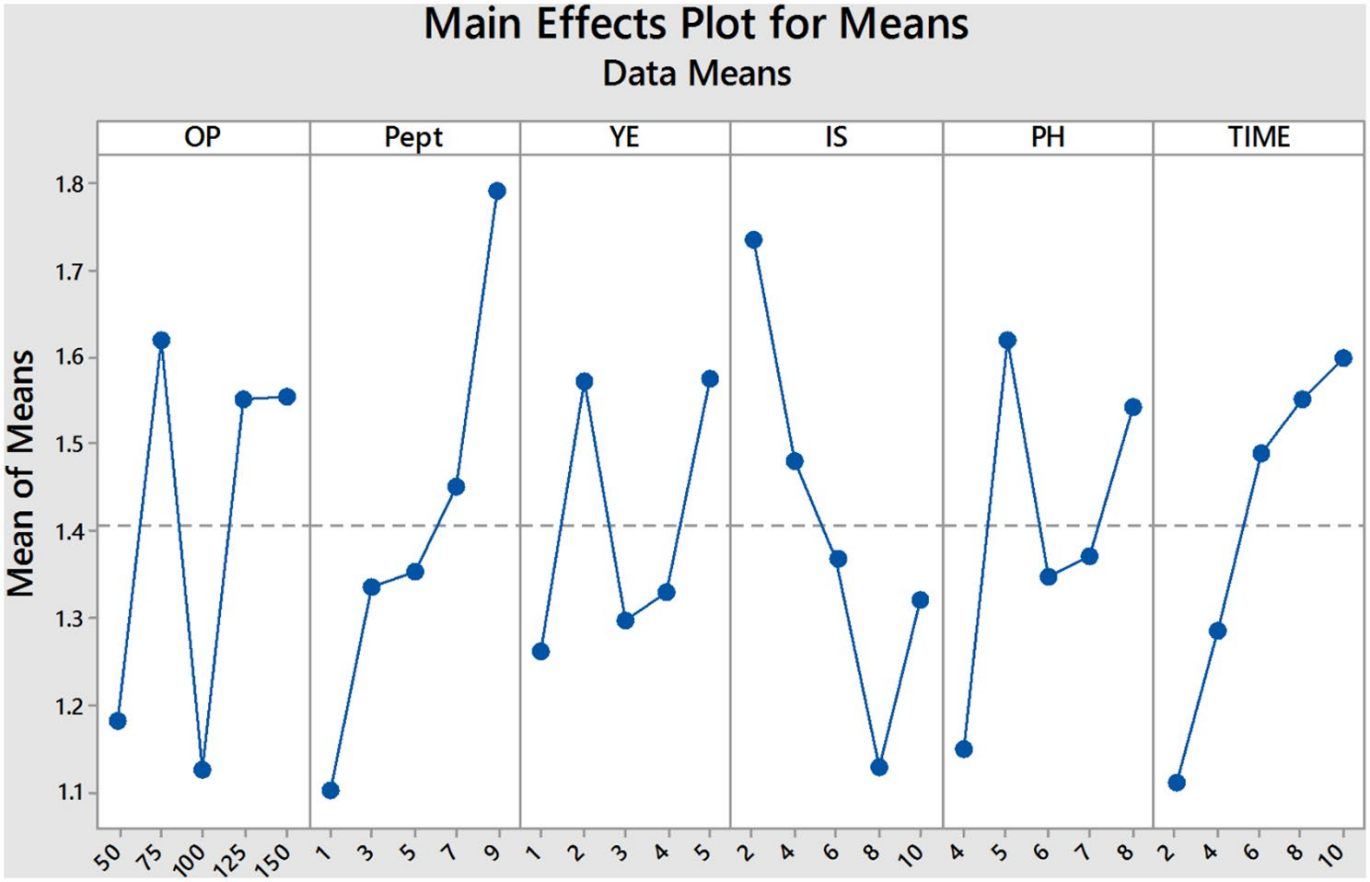
Main effects of orange peel, peptone, yeast extract, inoculum size, pH and incubation time on lipid production by *C. parapsilosis* Y19 strain

The Taguchi design can quantify the percentage of impact that each factor has on the lipid manufacturing process, allowing for the evaluation of their separate effects. The impact percentage for each factor was computed in relation to the lipid production value in this experiment. Table 1 presents the impact of all six factors that were studied. It shows that peptone has the greatest influence on lipid formation, with an effectiveness of 22.4%. Also, the effectiveness percentage for lipid production by Inoculum size, orange peel, incubation time, pH, and yeast extract were 19.7, 16.1, 15.9, 15.3 and 10.2%, respectively. Furthermore, the main effect plots of factors on the production shown in Fig. 4 confirms results in Table 4 where peptone is the highest factor which affect lipid production by *C. parapsilosis* Y19 followed by inoculum size. Also, yeast extract is the lowest factor affect lipid production.

**Table 4:** Response table for effects of tested factors on lipid production by *C. parapsilosis* Y19 strain

Level	Orange Peels (g/l)	Peptone (g/l)	Yeast extract (g/l)	Inoculum Size (%)	pH	Time (days)
1	1.18	1.1	1.26	1.736	1.148	1.108
2	1.62	1.336	1.572	1.48	1.62	1.284
3	1.124	1.352	1.296	1.368	1.348	1.488
4	1.552	1.452	1.328	1.128	1.372	1.552
5	1.556	1.792	1.576	1.32	1.544	1.6
Delta	0.496	0.692	0.316	0.608	0.472	0.492
Rank	3	1	6	2	5	4
Effectiveness %	16.124	22.496	10.273	19.766	15.344	15.997

**Fig. 5:**
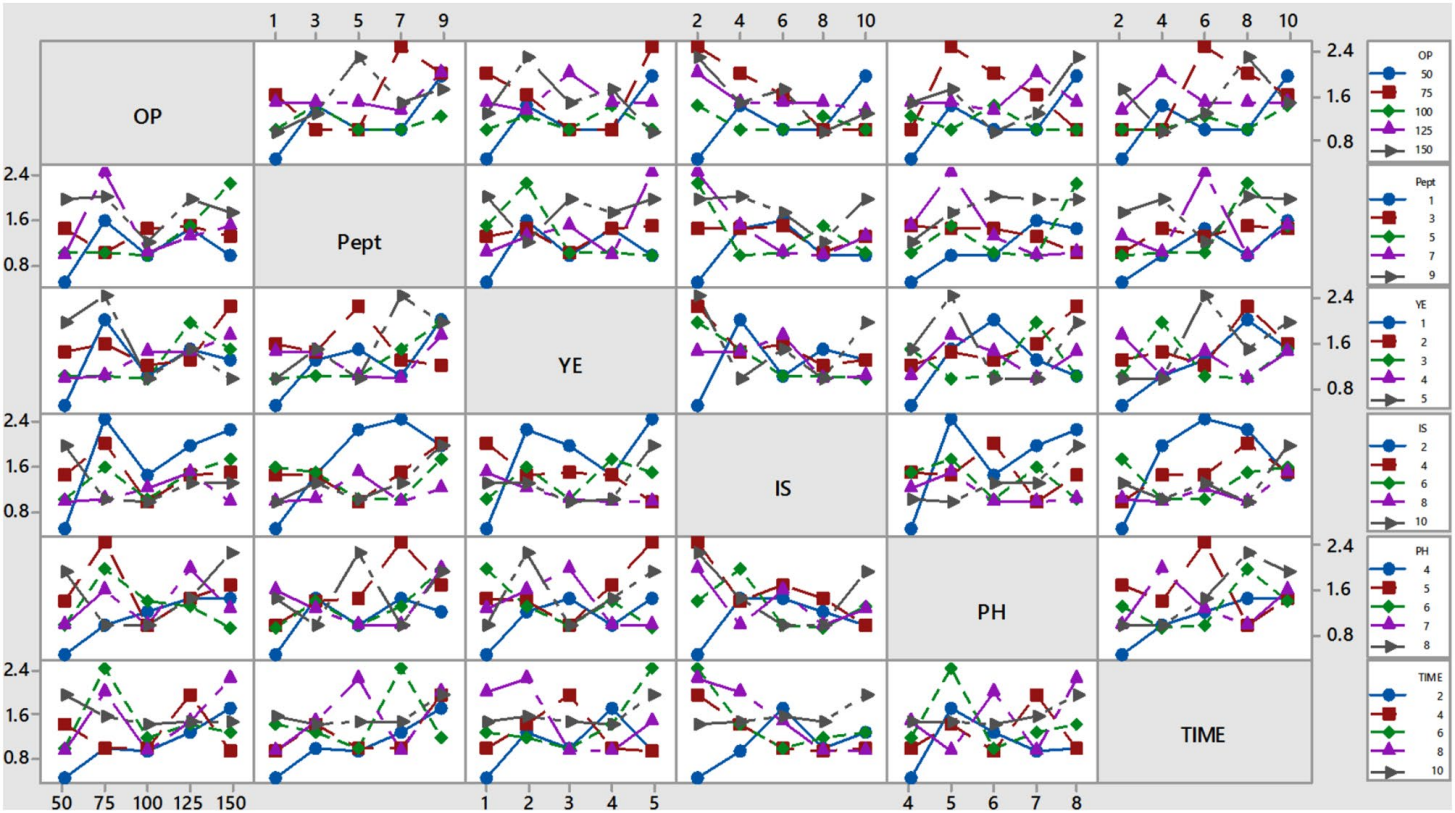
Interactions between different levels of orange peel, peptone, yeast extract, inoculum size, pH and incubation time for lipid production by *Candida parapsilosis* Y19 strain

The interactions between various levels of selected elements are crucial for determining the optimal level for each factor. The interaction between various levels of orange peel concentration and other parameters, revealing that an orange peel concentration of 75 g/l resulted in the highest lipid production (Fig. 5). In addition, the levels of peptone were combined with various levels of other components. It was shown that a yeast extract concentration of 7 g/l yielded the highest amount of lipid production. Additionally, the levels of yeast extract were examined in conjunction with various levels of other components. It was found that yeast extract concentration of 5 g/l yielded the highest lipid production, indicating that this was the optimal concentration. Furthermore, the size of the inoculum had a significant interaction with all levels of the other four parameters. It was shown that a 2% (*v/v*) inoculum size was the optimal for lipid production. When considered separately, both the levels of pH and incubation time showed interactions with various levels of other parameters. Specifically, a pH level of 5.0 and an incubation duration of 6 days were found to be the most favorable conditions for lipid production.

Thangavelu et al. [52] used sago processing wastewater as a substrate for growth of *Candida tropicalis* ASY2 for lipid production, where optimized conditions were 15.3 g/l of starch content, 0.5 g/l of yeast extract. Yong-Hong et al. [72] reported that, oleaginous yeast *Rhodosporidium toruloides* have ability to accumulate high lipid content with these optimal conditions: glucose 70 g/l, yeast powder 0.75 g/l, pH 6.0, inoculum 10% for 5 days at 30^o^C.

### Enhancement the lipid production by repeated batch fermentation

**Fig. 6:**
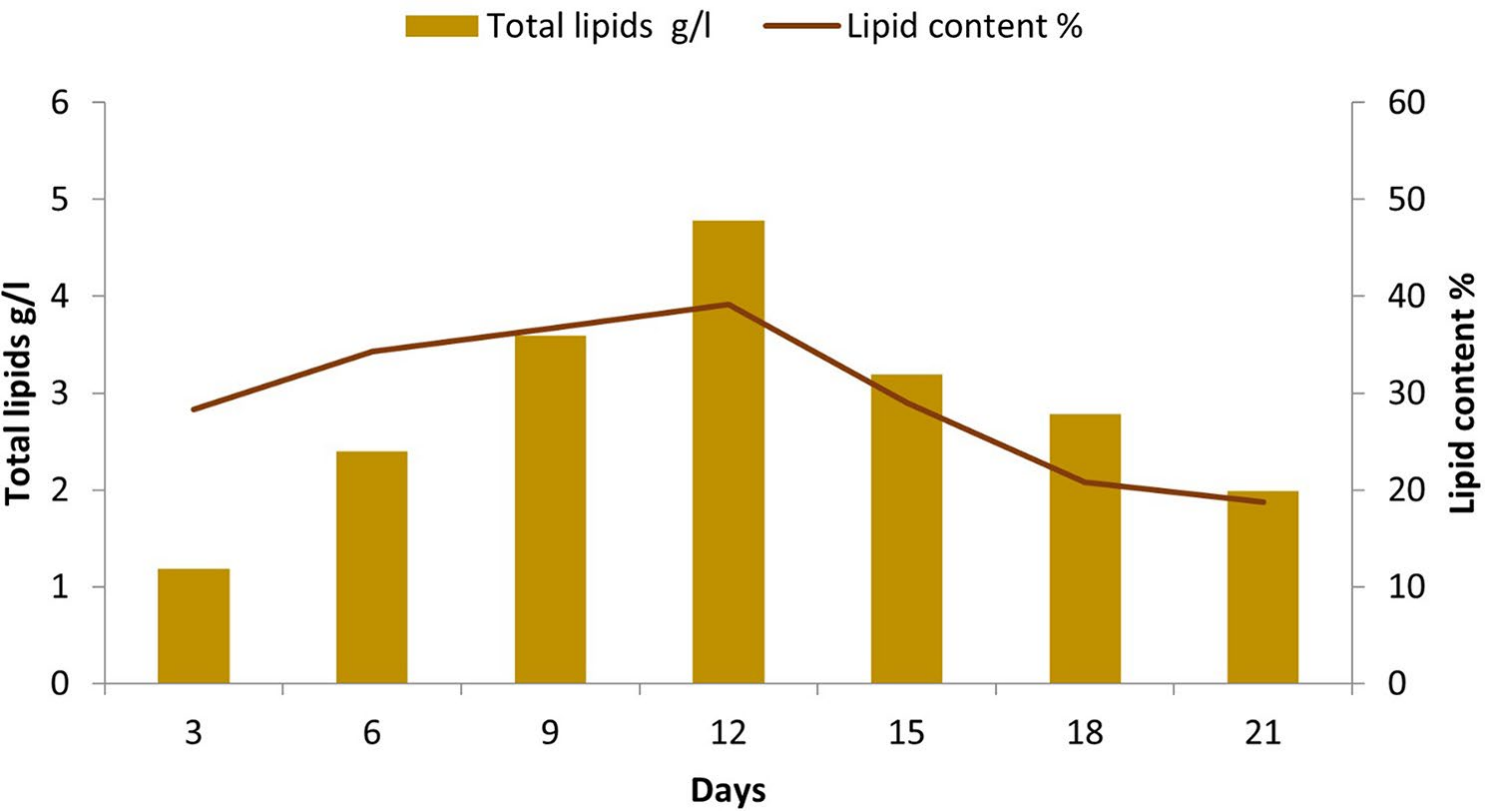
Lipid production from *C. parapsilosis* Y19 using repeated batch fermentation at different incubation times

Repeated batch fermentation is a fermentation process where the same fermentation vessel is used for multiple successive batches, with the addition of fresh substrate and inoculum between each batch [73]. Results indicated that repeated batch fermentation is a promising strategy for the production of lipids from *C. parapsilosis* that was conduced successfully for 7 runs. The maximum dried biomass, total lipids, and lipid content were 12.2 g/l, 4.78 g/l, and 39.1%, respectively obtained in the fourth run (Fig. 6). In their study, Dashti, Abdeshahian [74] highlighted the significance of both harvesting time and harvesting volume in determining the efficiency of repeated batch culture. Sriphuttha et al. [75] employed repeated-batch fermentation to produce lipids from *Rhodotorula paludigena *using crude glycerol as a substrate. The results of their study indicated that the biomass and lipid content were 38.2 g/l and 38.2%, respectively, after batch no. 4. Wang et al. [76] used repeated-batch fermentation to produce lipids from the oleaginous yeast *Trichosporon cutaneum* CX1 strain, which had a lipid content of approximately 30%.

### Comparison of different strategies used for lipid production from *C. parapsilosis* Y19

The enhancement of lipid production from oleaginous yeasts is important for the development of sustainable alternatives to fossil-based fuels and chemicals [77]. This can be achieved by employing various strategies, such as fermentation strategies and optimization of cultivation conditions. In the current study, different strategies were used; batch without optimization, batch with statistical optimization using RSM and repeated batch fermentation as shown in Table 5. The results of the batch fermentation of *C. parapsilosis* Y19 demonstrated that the dried biomass, total lipids, and lipid content were 4.95 g/l, 1.14 g/l, and 23.0%, respectively. Moreover, statistical optimization of cultural conditions of *C. parapsilosis* Y19 with batch fermentation increased the lipid quantity more than two folds (2.15 times; 2.46 g/l) and lipid content to 1.33 fold (30.7%) compared to batch culture. Furthermore, the repeated batch fermentation of *C. parapsilosis* Y19 was the best for lipid production where lipid quantity increased 4.19 folds (4.78 g/l) compared to batch culture. Also, the lipid content was increased by1.7 fold (39.1%) compared to batch culture.

**Table 5:** Lipid production from *C. parapsilosis*Y19 using different strategies

Strategy	Dry biomass g/l	Lipid g/l	Lipid content %
Batch fermentation	4.98 ± 0.125^c^	1.14 ± 0.035^c^	23.03 ± 0.115^c^
Batch fermentation using RSM	8.00 ± 0.086^b^	2.46 ± 0.043^b^	30.75 ± 0.229^b^
Repeated batch fermentation	12.20 ± 0.087^a^	4.78 ± 0.053^a^	39.18 ± 0.176^a^

Letters a, b & c mean significance power where *P*-value was less than 0.05. R-sq, R-sq(adj), R-sq(pred) were 99.94%, 99.92% & 99.87% for lipid g/l respectively

In comparison with various oleaginous yeast strains, *Candida parapsilosis* Y19 demonstrated notable lipid production and productivity under repeated batch fermentation in shake flasks using orange peel as the feedstock. Specifically, this strain achieved a biomass concentration of 12.2 g/l, with a lipid yield of 4.78 g/l, corresponding to a lipid content of 39.1% and a lipid productivity of 0.02 g/L/h. These values were compared with other yeast species cultivated under different modes and conditions. Table 6 show a comparison table including lipid productivity and other performance metrics (g/L/h) by various oleaginous yeasts.

**Table 6:** A comparison table including lipid productivity and other performance metrics by various oleaginous yeasts

Yeast strain	Feedstock	Culture mode	Cultivation mode	Biomass Conc. (g/l)	Lipid yield (g/l)	Lipid Content (%)	Lipid Productivity (g/L/h)	Ref
*Rhodotorula* *taiwanensis* AM2352	Corncob hydrolysate	5-L bioreactor	Batch culture	33.9	16.9	50.1	0.14	[83]
*Rhodotorula glutinis*	Molasses	shake flask	Batch	10.3	8.08	45.0	0.03	[84]
*R. toruloides*	Sugarcane molasses	1 L-bioreactor	Fed-batch	22.0	13.4	61.0	0.25	[85]
*S. pararoseus KM281507*	Crude glycerol	3-L bioreactor	Batch culture	10.6	3.26	30.7	0.02	[78]
*R. toruloides ATCC 10,788*	Crude glycerol	Shake flasks	Batch culture	21.1	11.2	53.2	0.06	[79]
*Y. lipolytica*	Crude glycerol	5-L bioreactor	Batch culture with two-stage cultivation using dissolved oxygen shift	25.8	13.6	52.7	0.20	[80]
*C. curvatus*	Raw Glycerol	shake flasks	Batch culture	4.50	1.25	27.7	0.01	[81]
*Candida viswanathii*	Raw glycerol	7-L bioreactor	Fed-batch culture	17.0	5.60	32.9	0.03	[82]
*Candida parapsilosis Y19*	Orange peel	Shake flasks	Repeated batch	12.2	4.78	39.1	0.02	This work

For instance, *S. pararoseus* KM281507 cultivated with crude glycerol yielded a biomass of 10.6 g/l and a lipid content of 30.7% in a 3-L bioreactor during batch culture, reaching a productivity of 0.02 g/L/h [78]. In shake flask batch culture, *R. toruloides* ATCC 10,788, using crude glycerol as the feedstock, achieved a substantially higher lipid yield of 11.2 g/l and lipid content of 53.2%, with a productivity of 0.06 g/L/h [79]. Meanwhile, *Y. lipolytica* cultivated in a two-stage cultivation using dissolved oxygen shift in a 5-L bioreactor using crude glycerol, reported a lipid yield of 13.6 g/l and lipid productivity of 0.20 g/L/h, with a 52.7% lipid content [80]. Furthermore, *C. curvatus*, under batch culture in shake flasks, using raw glycerol, yielded 4.5 g/l biomass with 27.7% lipid content and a productivity of 0.01 g/L/h [81]. Finally, *Candida viswanathii*, in fed-batch culture using a 7-L bioreactor with raw glycerol, recorded a lipid yield of 5.6 g/l, lipid content of 32.9%, and productivity of 0.03 g/L/h [82]. In a broader comparison with other oleaginous yeast strains, *Rhodotorula taiwanensis* AM2352, cultivated in a 5-L bioreactor with corncob hydrolysate as the feedstock, achieved a biomass concentration of 33.9 g/l, lipid yield of 16.9 g/l, lipid content of 50.1%, and lipid productivity of 0.14 g/L/h [83]. *Rhodotorula glutinis*, in shake flask batch culture using molasses as the feedstock, yielded a biomass of 10.3 g/l with 45% lipid content and a productivity of 0.03 g/L/h [84]. Similarly, *R. toruloides*, cultivated in a 1 L-bioreactor under fed-batch conditions with sugarcane molasses, showed a biomass of 22.0 g/l, lipid yield of 13.4 g/l, and lipid content of 61%, achieving a lipid productivity of 0.25 g/L/h [85].

### Fatty acid composition of the lipid produced from *C. parapsilosis* Y19

The fatty acid profile of fungal lipids is important in various biotechnological and medical applications. Fungi are known to produce a diverse array of lipids, including triacylglycerols, phospholipids, and sterol esters, which exhibit unique fatty acid compositions [86]. Additionally, the fatty acid profile of fungal lipids can influence their physical and chemical properties, impacting their suitability for use in biofuel production, food and feed additives, and the development of novel pharmaceutical and cosmetic formulations [87]. Understanding and exploiting the diverse fatty acid profiles of fungal lipids is, therefore, a crucial aspect of leveraging the biotechnological potential of these microbial resources [88]. In the current study, the most promising oleaginous *C. parapsilosis* Y19, which produced the highest quantity of lipids in the case of batch fermentation using RSM and repeated batch fermentation was analyzed for fatty acid profiles using GC-MS as shown in Table 7. Results revealed that, fatty acid profile of lipid produced from *C. parapsilosis* Y19 using batch fermentation with RSM showed the saturated fatty acids (SFAs) at 24.4% while unsaturated fatty acids (USFAs) were 75.5%. Likewise, fatty acid profile when using repeated-batch fermentation exhibited SFAs 25.1% and USFAs 74.8%. Moreover, total poly unsaturated fatty acids (PUFAs) of the produced lipid in the case of batch with RSM and repeated batch were 46.1 and 26.0%, respectively. Also, total MUFAs in the two strategies were 29.4 and 48.7%, respectively. Furthermore, in the batch with RSM, the dominant fatty acid among all fatty acids was linoleic acid with 46.1% followed by oleic acid 28.6%. Additionally, in repeated-batch fermentation, the major fatty acid was oleic acid with 45.0% followed by linoleic acid 26.0%.

**Table 7:** Fatty acid profiles of *C. parapsilosis* Y19 of lipids produced at batch with RSM and repeated-batch fermentations

Fatty acid	Type	Fatty acid (%) of lipids produced by:	
		Batch fermentation with RSM	Repeated batch fermentation
Myristic acid (C14)	SFA	--	0.30
Palmitoleic acid (C16)	MUFA	0.29	0.65
Palmitic acid (C16)	SFA	13.36	14.38
Cis-10 heptadecanoic acid (C17)	MUFA	0.56	2.62
Margaric acid (C17)	SFA	0.59	2.49
Linoleic acid (C18)	PUFA	46.10	26.03
Oleic acid (C18)	MUFA	28.61	45.04
Stearic acid (C18)	SFA	9.39	6.44
Arachidic acid (C20)	SFA	0.44	0.47
13-Docosenoic acid (C22)	MUFA	--	0.47
Behenic acid (C22)	SFA	0.29	--
Lignoceric acid (C24)	SFA	0.37	0.89
Cerotic acid (C26)	SFA	--	0.22
SFAs		24.44	25.19
USFAs		75.56	74.81
MUFAs		29.46	48.78
PUFAs		46.1	26.03

Plants are a typical source of linoleic acid (LA) which is especially beneficial when present in seed oils. The only important omega-6 fatty acid that needs to be consumed every day through diet is linoleic acid [89]. Elongase and desaturase enzymes can be used to convert linoleic acid into many other omega-6 fatty acids. As a result, linoleic acid acts as a precursor for the synthesis of other *n*-6 acyl species and important fatty acids such as arachidonic acid [90]. Thangavelu et al. [52] reported that, Fatty acid methyl esters (FAME) profile of lipid produced from *C. tropicalis* has oleic acid as a major fatty acid with 41.3%, but it contains linoleic acid with very low quantity 1.70%. Katre et al. [91] used cooking oil waste as substrate for lipid production from oleaginous yeast *Y. lipolytica*, where fatty acid profile showed oleic and linoleic acids with 25.5 and 30.6%, respectively. Horincar et al. [92] reported that, lipids from *Y. lipolytica* contain oleic and linoleic acids at 30 and 20%, respectively.

## Conclusion

This study demonstrates the potential of *Candida parapsilosis* Y19 as a promising oleaginous yeast for lipid production. The strain’s ability to efficiently utilize orange peel, a cost-effective and readily available substrate, highlights its adaptability and potential for sustainable biofuel production. Optimization through response surface methodology (RSM) and the implementation of repeated-batch fermentation significantly enhanced lipid yield, reaching 4.78 g/l and a lipid content of 39.1%. The repeated-batch fermentation strategy proved particularly effective, significantly increasing lipid production compared to batch culture. Furthermore, the favorable fatty acid profile of the produced lipids, rich in unsaturated fatty acids like oleic and linoleic acid, suggests their potential for diverse applications, including pharmaceuticals, biofuel production, and the synthesis of valuable oleochemicals. These findings emphasize the versatility and desirability of *C. parapsilosis* Y19 as a source for sustainable lipid production. This study demonstrates the potential of using waste-derived carbon sources, specifically orange peel, for sustainable lipid production, which supports both biofuel development and waste valorization. By substituting traditional carbon sources, orange peel reduces costs and aligns with environmental goals, offering a practical model for industrial microbiology. Future works should be focused on scaling and economically optimizing these processes, with potential applications including biodiesel production and the use of omega-3-rich linolenic acid in food and pharmaceuticals, highlighting oleaginous yeasts’ value in sustainable biotechnology.

## Data Availability

No datasets were generated or analysed during the current study.
